# The Effect of Additional Whole-Body Vibration on Musculoskeletal System in Children with Cerebral Palsy: A Systematic Review and Meta-Analysis of Randomized Clinical Trials

**DOI:** 10.3390/jcm12216759

**Published:** 2023-10-25

**Authors:** Márk Ágoston Pulay, Rita Nagy, Tamás Kói, Andrea Harnos, Nóra Zimonyi, Miklós Garami, Ákos Gasparics, Péter Hegyi, Ibolya Túri, Éva Feketéné Szabó

**Affiliations:** 1 Centre for Translational Medicine, Semmelweis University, 1085 Budapest, Hungary; nagyrita003@gmail.com (R.N.); samatiok@gmail.com (T.K.); harnos.andrea@gmail.com (A.H.); norazimonyi@gmail.com (N.Z.); hegyi2009@gmail.com (P.H.); 2 András Pető Faculty, Semmelweis University, 1125 Budapest, Hungary; turi.ibolya@semmelweis.hu (I.T.); feketene.szabo.eva@semmelweis.hu (É.F.S.); 3 Department of Ergonomics and Psychology, Faculty of Economic and Social Sciences, Budapest University of Technology and Economics, 1111 Budapest, Hungary; 4 Heim Pál National Pediatric Institute, 1089 Budapest, Hungary; 5 Institute for Translational Medicine, Medical School, University of Pécs, 7622 Pécs, Hungary; 6 Department of Stochastics, Institute of Mathematics, Budapest University of Technology and Economics, 1111 Budapest, Hungary; 7 Department of Biostatistics, University of Veterinary Medicine, 1078 Budapest, Hungary; 8 Pediatric Center, Semmelweis University, 1085 Budapest, Hungary; miklos.garami@gmail.com; 9 Department of Obstetrics and Gynecology, Semmelweis University, 1088 Budapest, Hungary; gasparics.akos@semmelweis.hu; 10 Division of Neonatology, Department of Pediatrics, Semmelweis University, 1083 Budapest, Hungary; 11 Institute of Pancreatic Diseases, Semmelweis University, 1085 Budapest, Hungary

**Keywords:** cerebral palsy, vibration therapy, intervention, motor development, mobility, musculoskeletal system

## Abstract

Nowadays, whole-body vibration (WBV) has become increasingly popular as an additional therapy in the intervention of patients with cerebral palsy (CP). However, the impact of WBV remains a subject of debate. Consequently, a systematic review and meta-analysis were undertaken to evaluate the effects of WBV on the musculoskeletal system in children with CP. Randomized controlled trials (RCTs) were sought in the most frequent databases. The intervention studied was WBV combined with conventional physiotherapy (PT) compared with conventional PT as the control; the main outcomes were changes in the musculoskeletal system. Weighted mean differences with 95%CIs were calculated. A random-effects model was applied, and the publication bias was checked using funnel plots. On the basis of the inclusion and exclusion criteria, 16 articles, including 414 patients, were considered in the final analysis. The improvement in walking performance (speed and step length) was statistically significant (*p* < 0.05), and although there were no significant differences in the further outcomes, a clear positive tendency was visible in the case of improved muscle strength, decreased spasticity, enhanced gross motor functions, and overall stability. Based on the findings, a clear assessment of the usefulness of this intervention cannot be made; nonetheless, due to the promising results, it would be worthwhile to conduct additional RCTs to enhance the available evidence in this field. Due to the wide range of vibration configurations, including varying durations and intensities, it is suggested to establish guidelines and a strategy for the incorporation of this additional treatment.

## 1. Introduction

Cerebral palsy (CP) is a movement disorder characterized by permanent damage to the development of posture and movement caused by non-progressive disturbances that occur during fetal or infant brain development [1]. It is the most common childhood physical disability and affects 2 to 2.5 children per 1000 born in Western countries [2,3]. Cerebral palsy can be classified by functional abilities and by neurological subgroups, which are determined according to the limbs affected (hemiparesis, tetraparesis, or diparesis), clinical signs and symptoms (spasticity, dyskinesia, or ataxia), and muscle tone (hypotonic or hypertonic) [4]. While there are several classification systems available, the Gross Motor Function Classification System-Expanded & Revised (GMFCS-E&R) is the most frequently used system, consisting of five levels, I (least impairment) to V (most severe impairment). This system provides a standardized classification that assists in prognosis, treatment, and effective communication among clinicians, researchers, parents, and caregivers [5]. 

Children with CP require management by a multidisciplinary team. The most common therapeutic interventions are physiotherapy (PT), conductive education, and occupational therapy aimed at improving muscle strength, normalizing tone, facilitating normal movement patterns, and increasing quality of life [6]. There are various other additional therapies that may enhance the efficacy of these interventions. One of these complementary approaches is whole-body vibration (WBV) [7]. Whole-body vibration is one of the techniques for muscle training that is frequently used in several clinical settings for patients with motor disorders such as CP. Whole-body vibration is described as exercising or standing on a vibrating surface that sends vertical sinusoidal oscillations that can be applied locally or through the feet into the entire body [8]. Vibration is a potent stimulus for musculoskeletal systems due to the necessity of rapidly adjusting muscle stiffness to suit the waves. This reaction is mediated by monosynaptic and polysynaptic afferent pathways capable of triggering various responses [9]. Changes in the hormonal profile and cardiovascular response have also been observed after the application of WBV, suggesting that the use of whole-body vibration may assist in improving factors related to CP [10]. Numerous systematic reviews have highlighted the potential of WBV for patients with CP, demonstrating its capacity to enhance various musculoskeletal functions, including gross motor function, bone density improvement, spasticity and contracture reduction, as well as the enhancement in balance and muscle strength [11,12,13,14].

The intensity of WBV depends on factors like amplitude, frequency, and oscillation magnitude [9,14]. Low-amplitude, low-frequency stimulation is thought to enhance muscle strength and potentially reduce spasticity and improve musculoskeletal parameters [9,15]. External factors, such as body positioning and exposure time, can influence vibration intensity [16]. A recent study compared a gradually increased 7–18 Hz WBV protocol with a static 11 Hz protocol in children with spastic CP. The 7–18 Hz protocol showed immediate improvements in spasticity, while the static 11 Hz protocol appeared superior after eight weeks, although both had similar effects on physical performance [17].

Whole-body vibration appears to play a considerable role in reducing spasticity and improving gait, balance, and motor function in stroke patients [18]. Nowadays, WBV has become increasingly popular as an additional therapy in treating CP patients as well [19,20]. However, the impact of WBV remains a subject of debate due to the diverse and sometimes conflicting research findings. The most favorable setting parameters and the long-term effects are still unclear. The strength of this work lies in its broad coverage, analyzing the most comprehensive set of outcomes available from the published and acceptable RCTs to date, regardless of CP subtype. Consequently, a systematic review and meta-analysis were undertaken to evaluate the effects of WBV on the musculoskeletal system and related functions such as mobility, balance, and gross motor functions in children with CP. 

## 2. Methods

### 2.1. Study Design

This research followed the Preferred Reporting Items for Systematic Reviews and Meta-Analyses (PRISMA) recommendations [21]. The PRISMA guideline was primarily created for systematic reviews evaluating the effects of health interventions, with flexibility in accommodating various study designs [22]. This study was registered on PROSPERO (CRD42021284999).

### 2.2. Eligibility Criteria

This systematic review and meta-analysis included only RCTs that examined the impact of WBV on the musculoskeletal system or its related aspects in children with CP regardless of subtype and GMFCS level. Primary outcomes were muscle strength and spasticity, gross motor function, bone density, and walking skills: speed, walking distance, and balance. These outcomes must be measured in a comparable manner, provided with numerical data. 

The PICO framework [23] was developed in order to perform an accurate search strategy. The population of children diagnosed with CP were compared, where one group received WBV in combination with conventional physiotherapy (PT), while the other group received a placebo, sham, or simulated intervention as a control alongside conventional PT. The main outcomes of interest were changes in the musculoskeletal system (mobility, balance, muscle strength, spasticity, muscle function, bone density, gross motor functions, gait speed/walking distance, and motor performance).

### 2.3. Databases and Search Strategy

Studies published before 1 November 2022 were retrieved from the following databases: MEDLINE (via PubMed), Embase, Cochrane Central Register of Controlled Trials, Scopus, and Web of Science. The following keywords were used for the search: (“cerebral palsy” OR paraplegia OR diplegia OR CP OR hemiparesis OR hemiplegia OR tetraplegia) AND (“whole body vibration” OR “whole-body vibration” OR WBV OR “vibration therapy” OR vibration). No restrictions or filters were applied. 

### 2.4. Selection of Studies

Two reviewers independently utilized reference management software (Clarivate Analytics. (2019). EndNote (Version X9.3.3) [Software]. Philadelphia, PA, USA) to conduct the selection in duplicate. First, duplicates were removed automatically and then manually. The records were selected by title, abstract, and full text according to a set of predetermined rules stated in data selection and extraction protocol. Disagreements between the two reviewers were resolved through consensus. After each step of the selection procedure, the agreement rate was calculated using the Cohen coefficient.

### 2.5. Data Extraction

Two reviewers (M.Á.P. and Z.N.) independently extracted data into a standardized data collection sheet (Microsoft Corp. (2018). Microsoft Excel 2019 [Software]. Microsoft. Redmond, WA, USA). Data collected included sex distribution, age distribution, type and severity of CP (if reported), patient numbers, and mean or median values of outcomes of interest. The WBV intervention and conventional PT control groups were set. 

### 2.6. Risk of Bias

The risk of bias was assessed using the revised Cochrane risk-of-bias tool for randomized trials (RoB 2) [24]. Two reviewers independently assessed the selected articles for the following domains: randomization process, deviations from intended interventions, missing outcome data, measurement of the outcome, and selection of the reported result. Inclusion criteria had been established for selecting two independent evaluators: expertise in the field, trained in risk-of-bias assessment, pilot testing. Consequently, the studies were classified based on their risk of bias as either low, high, or with some concerns. Any disagreement was resolved through the consensus of the authors.

### 2.7. Statistical Analysis

Statistical analyses were carried out by using the package ‘meta’ in the R statistical software (R Core Team. (2021). meta: General Package for Meta-Analysis (Version 4.1.2) [Software]. R Foundation for Statistical Computing. Vienna, Austria). The statistical analyses followed the advice of Harrer et al. [25]. 

The efficiency of WBV was assessed by analyzing the mean and standard deviation of changes in various outcome measures before and after treatment in both the WBV intervention group and the control group, albeit with certain limitations outlined in the Limitations section. Subsequently, a random-effects meta-analysis was employed to evaluate the differences in mean changes. The classical inverse variance method with the restricted maximum likelihood estimator was utilized for this analysis. As only a few studies contributed to the meta-analysis, the Hartung–Knapp adjustment was applied. Besides the prediction interval, heterogeneity was assessed by calculating the I^2^ measure and its confidence interval and performing the Cochrane Q test. I^2^ values of 25%, 50%, and 75% were considered low, moderate, and high heterogeneity, respectively. It should be noted that a positive pooled value indicates that the change in the treatment group was larger. 

Whenever possible, the correlations between the values of the outcome before and after the treatments were calculated using raw data received from the authors or using published summary statistics. The average of the calculated correlations was used to estimate the standard deviation of the change when it was missing. Based on the subtype and GMFCS categories, subgroup analyses were planned to be performed.

## 3. Results

### 3.1. Qualitative Synthesis of Studies

**Figure 1:**
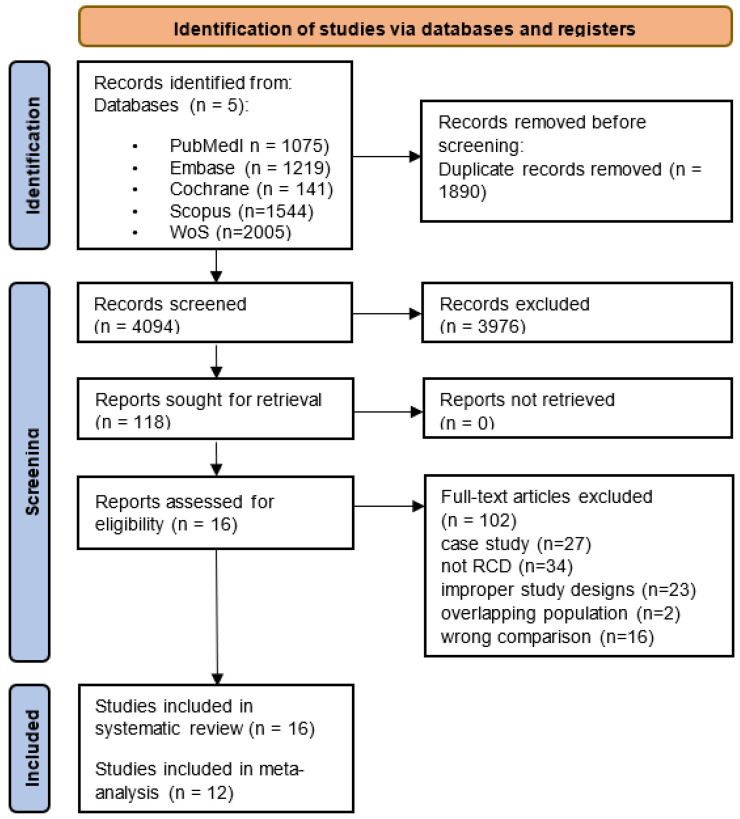
PRISMA 2020 flow diagram.

A total of 5984 potentially relevant records were identified in the databases. After removing 1890 duplicates, 4094 titles and abstracts were read, of which 3976 were excluded for not meeting the eligibility criteria. Out of the remaining 118 studies, 102 were not retrieved, following full-text selection. This was primarily due to their not being RCTs, having inappropriate study designs (such as different comparisons among observed groups), or involving overlapping patient populations. On the basis of the inclusion and exclusion criteria, 16 articles, including 414 patients, were considered in the final analysis. A total of 16 studies were considered eligible for inclusion in the systematic review, and 12 of them were integrated into the meta-analyses. The remaining four studies either employed special or unusual measurement techniques or did not report the statistical results appropriately. The PRISMA flowchart illustrating this process is displayed in Figure 1. 

Comprehensive details concerning the articles included in the systematic review can be found in Table 1, where all relevant characteristics have been compiled. The 16 reports included in this systematic review were published between the years 2010 and 2022. 

The mean age ranged from 19 months to 11.82 years. In reference to the further characteristics of the studies’ patients, certain studies exclusively present data concerning the Gross Motor Function Classification System (GMFCS) level of the subjects, encompassing a range from level I to IV. Alternatively, some studies report the specific type of CP, such as hemiplegia, diplegia, or tetraplegia. Furthermore, there are some studies that provide comprehensive information by reporting both GMFCS levels and CP types concurrently. For a detailed tabulation of this data, please look at Table 1.

The research papers employed two primary types of randomized study designs: Randomized clinical trials, where subjects were divided into two groups. The intervention group received WBV in conjunction with conventional physiotherapy (PT), while the control group received a placebo, sham, or simulated intervention alongside conventional PT or conventional PT only as a control. Another frequently utilized method was the crossover study design, where subjects were randomly allocated to either the AB or BA sequence (A treatment/B intervention group). The conventional PT used in the studies for children with CP has been shown to improve muscle strength, local muscular endurance, and overall joint range of motion, and to contain neurodevelopmental techniques, proprioceptive training, and balance training.

The outcomes measured in the selected studies cover a wide spectrum, most of them related to the musculoskeletal system. Gross motor function was mostly measured using GMFM-66 or 88 (GMFM-66 or 88). Spasticity was measured using the Modified Ashworth Scale (MAS). Walking ability was measured using different tests such as the 1-Minute Walk Test (1 MWT), 6-Minute Walk Test (6 MWT), and Time-Up-and-Go Test (TUG). All outcome-measured methods are displayed in Table 1. 

In the context of biomechanical parameters related to WBV exercises and devices, there is a considerable range of variation (see Table 1). Most of the studies employ either side-alternating or vertical platforms with frequencies spanning from 5 to 40 Hz and varying exercise durations, ranging from 30 s to 10 min. The most frequently utilized intensity regimen consists of 3 sets of 3 min each, with 3 min of rest between each set. Concerning body positioning, the majority of studies implemented muscle-strengthening exercises targeting the lower limbs while utilizing WBV.

### 3.2. Therapeutic Effect

#### 3.2.1. Muscle Strength

**Figure 2:**
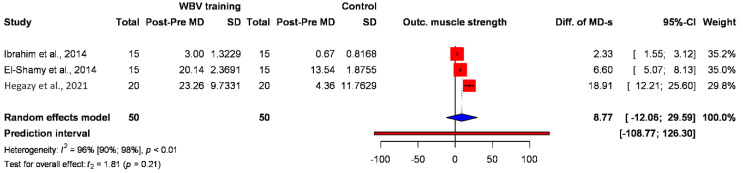
Forest plot with the pooled value of the difference in the MDs showing the effect of whole-body vibration (WBV) on muscle strength in intervention and control groups [8,30,31].

Four studies [8,29,30,38] assessed muscle strength as an outcome, and three of them measured the knee extensors with a dynamometer in Newton (N). The result between WBV training and the control group is statistically nonsignificant (diff. of MD 8.77) (95% CI: −12.06; 29.59); however, a positive pooled MD in changes between the treatment groups can be captured (Figure 2). 

#### 3.2.2. Spasticity

Five studies [26,28,29,35,36] assessed spasticity using the Modified Ashworth Scale (MAS), known as a clinical measure of muscle spasticity in people with neurological conditions [39]. MAS scores were given numeric values for evaluation (0, 1, 2, 3, 4, and 5) [36]. 

**Figure 3:**
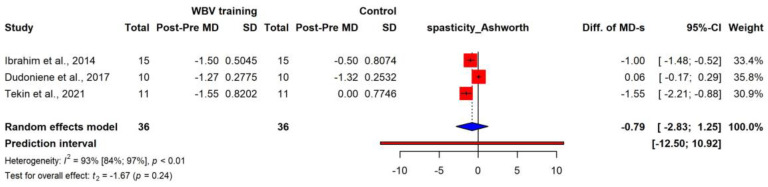
Forest plot with pooled value of the difference in the MDs representing the change in spasticity (measured with Ashworth scale) between intervention and control groups [29,31,35].

Of the four studies, three were included in the statistical analysis due to missing data. All studies reported increased muscle tone of the lower extremities; however, the pooled effect did not reach the level of significance (diff. of MD −0.79) (95% CI: −2.83; 1.25) (Figure 3).

#### 3.2.3. Gross Motor Function

**Figure 4:**
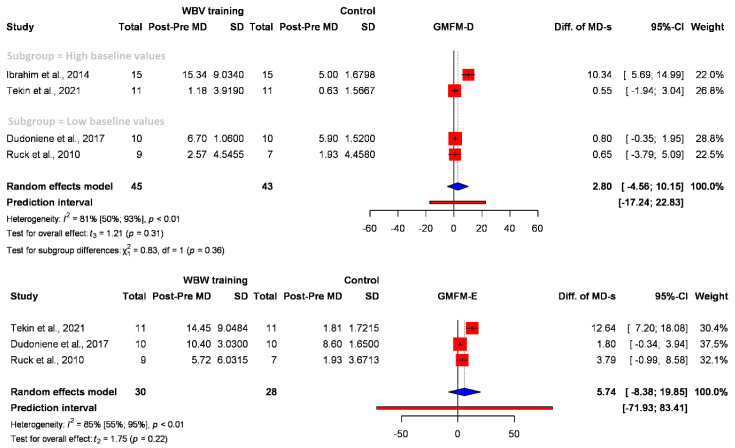
Forest plots displaying the efficacy of whole-body vibration (WBV) on standing (GMFM D-domain), and walking, running, and jumping (E-domain) between intervention and control groups [29,31,33,35].

The Gross Motor Function Measure (GMFM) measures the performance of the child in five dimensions, and each dimension can be measured separately or together [40]. A statistical analysis was feasible only for domains D (standing) and E (gait activities) due to limited overlap in the domains assessed by individual studies, as depicted in Figure 4. Most of the studies reported positive changes in gross motor functions, but the pooled effect size was finally nonsignificant (D domain: (MD 2.80) (95% CI: −4.56; 10.15), E domain: (MD 5.74) (95% CI: −8.38; 19.85)). Importantly, the study conducted by Tekin et al. [35] demonstrated minimal change following the intervention. Nonetheless, it is noteworthy that the baseline GMFMD value of the included patients closely resembles that of typically developing children.

#### 3.2.4. Walking Performance

**Figure 5:**
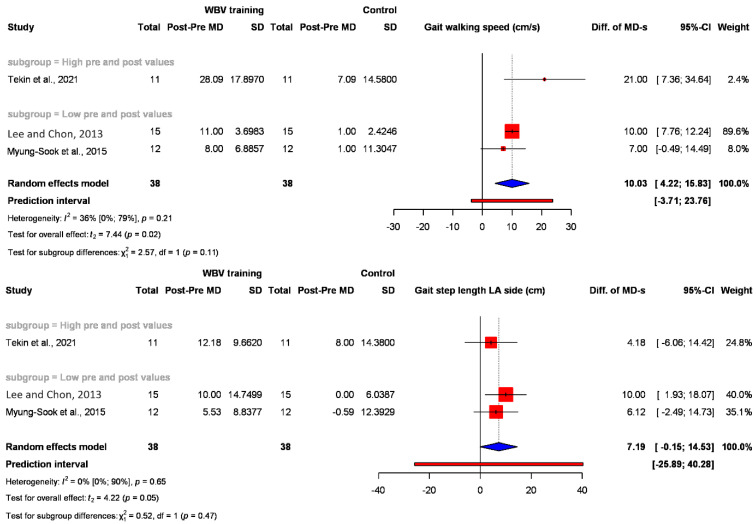
Forest plot with the pooled value of the difference in MDs showing the walking-related outcomes (walking speed and step length) in the intervention and control groups [7,32,35].

The effects of WBV treatment on walking skills were assessed not only through the GMFM E domain but also in various ways such as walking speed, step length, the 6-Minute Walk Test (6 MWT) [41], and the Time-Up-and-Go Test (TUG) [42]. Figure 5 shows two walking outcomes: walking speed and step length. Significant changes were found in two strongly related outcomes: the difference in the changes in walking speed (cm/s) (diff. of MD 10.03) (95% CI: 4.22; 15.1583) (*p* = 0.02) and step length (cm) (diff. of MD 7.19) (95% CI: −0.15; 14.53) (*p* = 0.05). The two other walking-related outcomes were nonsignificant: 6 MWT (meter/6 min) (diff. of MD 59.55) (95% CI: −164.26; 283.36) (Figure S2 in the Supplementary Materials) and TUG (test completed in sec) (diff. of MD −2.88) (95% CI: −10.37; 4.62) (Figure S3 in the Supplementary Materials).

#### 3.2.5. Overall Stability

Four studies [8,26,35,43] have assessed the effect of WBV on overall stability or balance. However, they all used several measurement methods, making the statistical analysis not feasible. Tekin and Kavlak [35] evaluated the balance skills of the participants using a SportKAT 550 tm portable computerized kinesthetic balance device. They revealed that the balance scores of the WBV group significantly improved after treatment compared with pretreatment (*p* = 0.03) and that the improvements were maintained after 12 weeks (*p* = 0.184). Ko et al. [43] reported that static postural balance improved following WBV interventions. However, the changes were not statistically significant for either the WBV or the control group. El-Shamy [8] observed that the overall stability index after treatment was 2.75 and 2.2 for the control and experimental groups, respectively. The mean values of the stability index increased in both groups, but the experimental group experienced a significant increase compared to the control group (*p* < 0.001). Aslam and Baig [27] used the pediatric balance scale [44], and they found statistically significant (*p* < 0.001) changes between the intervention and control groups.

### 3.3. Secondary Outcomes

#### 3.3.1. Muscle Thickness

Two studies investigated the positive effects of WBV on muscle thickness. Lee and Chon [7] involved 30 patients with CP in their RCT. They found that the experimental group had significantly thicker tibialis anterior (*p* = 0.001, 0.48 (0.08) mm to 0.63 (0.10)) and soleus (*p* = 0.001, 0.45 (0.04) mm to 0.63 (0.12)) muscles than the control group. However, no significant effect was observed on gastrocnemius muscle thickness (*p* = 0.645) [7]. Another study [15] reported that post-treatment values demonstrated significant improvement in the parameters in favor of the experimental group (*p* < 0.05), as there was an improvement in the thickness of the four abdominal muscles when compared to the control group (external oblique: F = 38.783, internal oblique: F = 99.547, transverse abdominis: F = 111.557, and rectus abdominis: F = 129.940, *p* < 0.05).

#### 3.3.2. Bone Density

Since the last systematic review [11], none of the new RCTs investigated the effect of WBV on bone density. They reported a significant improvement of 1.32 (95% CI: 0.28, 2.36, *n* = 47) for participants in the WBV group compared with the control group. They found a nonsignificant improvement of 0.41 in lumbar spine bone density (95% CI: −0.42, 1.25, *n* = 77) [11]. 

### 3.4. Risk of Bias

The included studies were assessed for the randomization process, deviations from intended interventions, missing outcome data, measurement of the outcome, and selection of the reported result. The evaluations of the risk of bias (RoB) for each outcome are summarized in Supplementary Figure S1. Biases were mostly assessed as “some concern”, primarily due to the selection of the reported result. Overall, the judgments predominantly indicated a “low risk”.

## 4. Discussion

In this systematic review and meta-analysis, the short- and long-term effects of combined whole-body vibration and conventional physiotherapy were investigated on the musculoskeletal system in children with cerebral palsy (CP). The meta-analysis revealed a significant difference in walking speed and step length, and most investigated outcomes had favorable tendencies.

Children with cerebral palsy have reduced muscle strength and control, limiting their ability to perform functional tasks such as standing and walking [36]. Four studies [8,29,30,38] assessed muscle strength as an outcome, and three of them measured the knee extensors using a dynamometer in Newton (N). The result between WBV training and the control group was statistically nonsignificant; however, a positive tendency could be observed. Gait speed and muscle strengthening in patients with cerebral palsy are known to be strongly correlated [28,45]. Statistically significant changes were found in the gait-speed-related outcome, and it supports this finding. Muscle strengthening training is one of the most important goals to prepare incapacitated muscles responsible for debilitated walking capacity, like the quadriceps muscle in patients with CP. Consequently, maintaining and enhancing lower extremity strength and force are essential factors for reducing functional limitations and the incapacity of patients with CP. WBV exercise increases muscle strength and force, which may lead to improvements in neuromuscular capacities.

Abnormal—either increased or reduced—muscle tone is one of the most significant symptoms in CP; it makes movement difficult or even impossible. Five studies [26,28,29,35,36] assessed spasticity using the Modified Ashworth Scale, which is known as a clinical measure of muscle spasticity in people with neurological conditions [39]. There were no significant differences between the two groups after interventions in terms of spasticity. However, a positive tendency was found for a slight decrease in tone. Studies that measured this outcome and conducted follow-ups after treatment reported that WBV treatment might decrease spasticity in patients with CP temporarily rather than permanently. Another study [28] reported that the effects of decreased tone could last up to three days after the intervention. 

Gross motor function is a frequently measured outcome; almost half of the selected studies applied the Gross Motor Function Measure [40] to determine the functional skill levels of the participants. All these studies reported positive changes, with most of them being statistically significant [11,30,34] improvements in one or two domains or total GMFM scores. The results also show remarkable (difference in the changes in D-domain, MD 2.80, and E-domain, MD 5.74) although statistically nonsignificant changes between WBV training and control groups. According to a study by Wang and Yang [46], a change score of 1.3 points on the GMFM-88 indicates a clinically relevant improvement, and a change score of 3.7 points distinguishes between great and moderate improvement. 

The effects of WBV treatment on walking abilities were evaluated not only using the GMFM E domain (gait activities) but also with several other methods, including walking speed, step length, 6-Minute Walk Test [41], and Time-Up-and-Go-Test [42]. On the one hand, this variety is advantageous because it has been demonstrated in multiple ways that WBV therapy improves walking ability. On the other hand, the fact that the same expected outcome has been tested in different ways makes statistical analysis challenging. All studies reported positive changes in walking parameters, and statistically significant changes were found in two assessed outcomes: walking speed (*p* = 0.02) and step length (*p* = 0.05). Another related outcome is that the overall stability or balance was assessed in three studies, but they applied different measurement methods, which made the statistical analysis impossible. Two of them reported a statistically significant improvement in balance skills in favor of the WBV group.

Since the last systematic review and meta-analysis published seven years ago in 2015 [11], there has been a notable increase in the number of RCTs conducted on this topic. In the prior analysis, only six studies were included, whereas in the current study, sixteen have been incorporated. The expanded dataset facilitates a more robust evaluation of the effects, encompassing the majority of the previously mentioned outcome measures, including walking- and gross-motor-function-related skills.

### Limitations of the Review Process

In the process of pooling relevant data, efforts were made to minimize the level of heterogeneity.

The following phenomena made the meta-analysis challenging: inconsistent or insufficient data reporting, different measurement methods, undefined patient population (unpublished GMFCS level), a small number of participants, and various intervention designs. Subgroup analyses based on CP subtypes or GMFCS categories were originally planned, but the heterogeneity of the study population and the limited number of studies on the assessed outcomes made it unfeasible.

The correlation imputation and the estimation of the mean and its standard deviation using the quartiles are widely accepted [24] in meta-analyses, as they only provide estimations of the true values, and their usage is also a limitation. 

## 5. Conclusions

### 5.1. Implications for Practice

In this systematic review and meta-analysis, we comprehensively examine and aggregate all available and relevant CP-related outcomes to assess the impact of WBV therapy. Significant changes were found in two walking-related outcomes (walking speed and step length) and nonsignificant although clinically important, positive improvements were found in many outcomes related to the musculoskeletal system. The assumed observation is that patients with milder CP (GMFCS I-II) show a more robust improvement after WBV therapy. This finding is supported by some of the significant results, mainly related to walking and balance.

According to the available data, the results indicate that WBV should be considered as an additional treatment to conventional physiotherapy to normalize muscle tone before functional therapy and improve gait functions and mobility in children with CP. 

### 5.2. Implications for Reasearch 

Based on the findings, a clear assessment of the usefulness of this intervention cannot be made; nonetheless, due to the promising results, it would be worthwhile to conduct additional RCTs to enhance the available evidence in this field. Due to the wide range of vibration configurations, including varying durations and intensities, it is suggested to establish guidelines and a strategy for the incorporation of this additional treatment. A limited number of studies included severely impaired CP patients, and none of them involved patients at the GMFCS V level. Exploring the impact of this additional therapy on the most severe CP cases could represent a valuable direction for future research. 

## Figures and Tables

**Table 1:** Characteristics of the articles included in the systematic review.

	Patient Characteristic				Groups		Outcome	Intervention Setup
Study	Type of CP	GFFCS Level	*n*	Age	Treatment	Control	Outcome Measures	Frequency (Times/Week) Length (Week) Intensity
Ahmadizadeh et al., 2019 [26]	S. Hemiplegia (*n* = 9) S. Diplegia (*n* = 10) Tetraplegia (*n* = 1)	I, II, III	20	7.5 years SD ± 2.23	Conventional Therapy + WBV	Conventional Therapy	Goniometry, spasticity, 6 MWT, ROM	2 × 3 min 3 times/week 6 weeks 20–24 Hz
Ali MS and Abd El-aziz HG 2020 [15]	S. Diplegia	I, II, III, IV	30	5.23 years SD ± 0.96	Conventional Therapy + WBV	Conventional Therapy	GMFM-88 sitting domain, abdominal muscle thickness ultrasonography	2 × 5 min 3 times/week 12 weeks 30 Hz
Aslam and Baig 2022 [27]	NR	II, III	38	9.36 years SD ± 1.26	Conventional Therapy + WBV	Conventional Therapy	MAS spasticity, manual muscle testing, pediatric balance scale, CPQOL	1 × 3 min 3 times/week 4 weeks 40 Hz
Cheng et al., 2015 [28]	S. Diplegia (*n* = 11) S. Quadriplegia (*n* = 5)	NR	16	9.2 years SD ± 2.1	Conventional Therapy + WBV	Conventional Therapy	AROM, PROM, RI, MAS spasticity, TUG, 6 MWT	1 × 10 min 3 times/week 8 weeks 20 Hz
Dudoniene et al., 2017 [29]	S. Diplegia	NR	20	8.60 years SD ± 0.96	Conventional Therapy + WBV	Conventional Therapy	Spasticity, range of motion, GMFM-88	5–10 min 5 times/week 3 weeks 15 Hz
El-Shamy 2014 [8]	S. Diplegia	I, II	30	9.79 years SD ± 1.13	Conventional Therapy + WBV	Conventional Therapy	Knee extensor strength, stability index	3 × 3 min 5 times/week 12 weeks Vibraflex Home Edition II. 12–18 Hz
Hegazy et al., 2021 [30]	S. Hemiplegia	I, II	40	6.95 years SD ± 1.46	Conventional Therapy + WBV	Conventional Therapy	Quadriceps, hamstring muscle strength, endurance, 6 MWT and power	3 × 3 min 3 times/week 8 weeks 10–25 Hz
Ibrahim et al., 2014 [31]	S. Diplegia	NR	30	9.93 years SD ± 1.41	Conventional Therapy + WBV	Conventional Therapy	Knee extensor strength, walking speed, walking balance, gross motor function	3 × 3 min 3 times/week 12 Week 12–18 Hz Power Plate
Lee and Chon 2013 [7]	S. Diplegia S. Hemiplegia	NR	30	9.83 years SD ± 2.39	Conventional Therapy + WBV	Conventional Therapy	Gait analyses and ultrasonographic imaging of the leg muscles	6 × 3 min 3 times/week 8 weeks 5–25 Hz
Myung-Sook et al., 2015 [32]	S. Diplegia (*n* = 14) S. Hemiplegia (*n* = 10)	I, II, III	24	9.52 years SD ± 2.38	Conventional Therapy + WBV	Conventional Therapy	Gait analyses, TUG test, Functional Independence Measure for Children (WeeFIM)	3 × 3 min 2 times/week 3 weeks 20–24 Hz Galileo
Ruck et al., 2010 [33]	NR	II, III, IV	20	6.2 to 12.3 years	Conventional Therapy + WBV	Conventional Therapy	Walking ability, bone densitometry, gross motor function	3 × 3 min 5 times/week 24 weeks 12–18 Hz Galileo
Stark et al., 2016 [34]	NR	II, III, IV	24	19 months SD ± 3.1	Conventional Therapy + WBV	Conventional Therapy	GMFM-66, PEDI,	3 × 3 min 10 times/week 14 weeks 12–22 Hz
Tekin and Kavlak 2021 [35]	S. Hemiplegia	NR	22	11.82 years SD ± 3.55	Conventional Therapy + WBV	Conventional Therapy	Gait analysis, standing, walking, balance, spasticity, gross motor function	1 × 15 min 3 times/week 8 weeks 15 Hz Compex-Winplate
Tupimai et al., 2016 [36]	NR:	I, II, III	12	10.6 years SD ± 2.4	Conventional Therapy + WBV	Conventional Therapy	MAS spasticity, PEDI, muscle strength	10 × 1 min 5 times/week 6 weeks 20 Hz
Unger et al., 2012 [37]	S. Diplegia S. Hemiplegia	I, II, III	27	6–13 years	Conventional Therapy + WBV	Conventional Therapy	1 MWT, 2D-posturography, ultrasound imaging and sit ups in one minute	30–40 s 5 times/week 4 weeks 35–40 Hz
Wren et al., 2010 [38]	S. Diplegia (*n* = 18) S. Hemiplegia (*n* = 4) S. Tetraplegia (*n* = 9)	I, II, III, IV	31	9.4 years SD ± 1.4	Conventional Therapy + WBV	Conventional Therapy	Bone density, plantar flexor strength	1 × 10 min 10 min/day 6 months (at home) 30 Hz

Abbreviations: NR, not reported; S, Spastic; WBV, whole-body vibration; GMFCS, Gross Motor Function Classification; GMFM (66 or 88), Gross Motor Function Measure; PEDI, Pediatric Evaluation of Disability Inventory; MAS, Modified Ashworth Scale; 1 MWT, 1-Minute Walk Test; 6 MWT, 6-Minute Walk Test; TUG, Time-Up-and-Go Test; (P/A) ROM, (Passive/Active) Range of Movement; CPQOL, Cerebral Palsy Quality of Life Questioner.

## Data Availability

The data presented in this study are available in this article and Supplementary Materials.

## Supplementary Materials

The following supporting information can be downloaded at https://www.mdpi.com/article/10.3390/jcm12216759/s1. Figure S1: Risk of bias assessment summary of the included studies. Figure S2: Forest plot with pooled value of the difference in the mean differences showing one of the walking-ability-related outcomes, 6-Min Walk Test, in the intervention and control group. Figure S3: Forest plot with pooled value of the difference in the mean differences showing one of the walking-related outcomes, TUG (Time-Up-and-Go Test), in the intervention and control group.
