# Supplementary material for: The Effect of Additional Whole-Body Vibration on Musculoskeletal System in Children with Cerebral Palsy: A Systematic Review and Meta-Analysis of Randomized Clinical Trials
Source: J Clin Med. 2023 Oct 25;12(21):6759. doi: 10.3390/jcm12216759 (PMC10648882; doi:10.3390/jcm12216759)
Supplement: Supplementary file 1 [file jcm-12-06759-s001.zip › jcm-2653579-supplementary.pdf]

Supplementary Materials

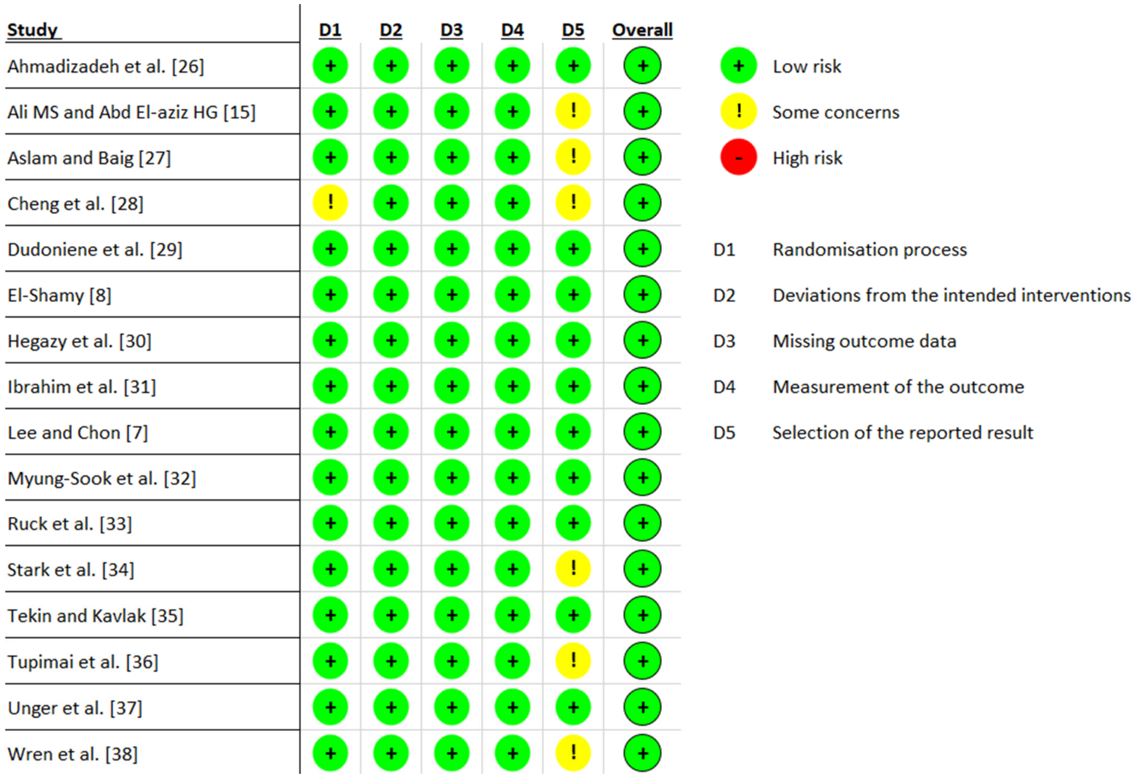

Figure S1. Risk of Bias assessment summary of the included studies.

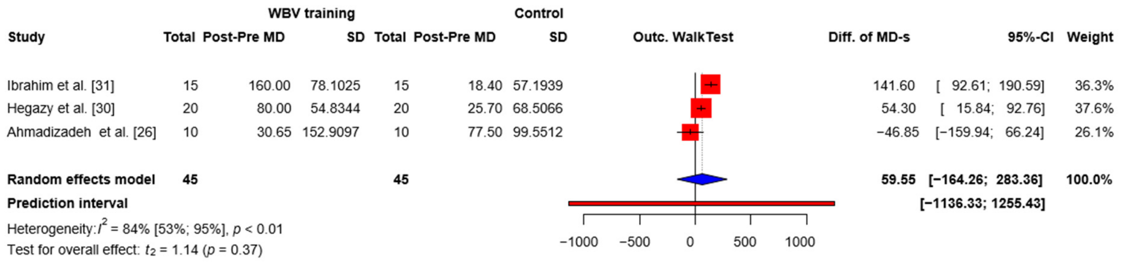

Figure S2. Forest plot with pooled value of the difference of the mean differences showing one of the walking ability-related outcomes, **6 Minute Walk Test** in the intervention and control group.

Study	WBW training Total	WBW training Post-Pre MD	WBW training SD	Control Total	Control Post-Pre MD	Control SD	Diff. of MD-s	95%-CI	Weight
Ibrahim et al. [31]	15	−2.33	7.1861	15	−3.00	3.3819	0.67	[−3.35; 4.69]	29.8%
Myung-Sook et al. [32]	12	−6.58	9.7305	12	−0.63	1.2574	−5.95	[−11.50; -0.40]	21.0%
Tekin and Kavlak [35]	11	−3.30	2.3305	11	0.41	1.0930	−3.71	[−5.23; -2.19]	49.2%
**Random effects model**	**38**			**38**			**−2.88**	**[−10.37; 4.62]**	**100.0%**
**Prediction interval**								**[−38.03; 32.28]**	

Heterogeneity: $I^2 = 59\%$ [0%; 88%], $p = 0.09$
Test for overall effect: $t_2 = -1.65$ ($p = 0.24$)

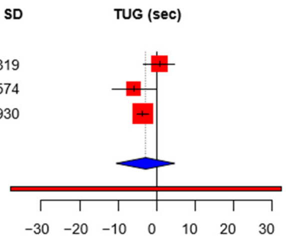

Figure S3. Forest plot with pooled value of the difference of the mean differences showing one of the walking-related outcomes, **TUG, Time Up and Go Test** in the intervention and control group.
